# Striving Against Sonlessness: The Moral Uses of Medical Pluralism in Western Indian Quests for a Boy

**DOI:** 10.1007/s11013-024-09880-6

**Published:** 2024-09-25

**Authors:** Utpal Sandesara

**Affiliations:** General Internal Medicine (School of Medicine) and Global Health (International Institute), University of California - Los Angeles, 1100 Glendon Avenue, Suite 900-912, Los Angeles, CA USA

**Keywords:** Medical pluralism, Morality, Reproduction, Sex selection, India

## Abstract

Amid patriarchal conditions that render one son necessary and multiple daughters burdensome, selective abortion of female fetuses has become pervasive in India. Public responses often cast sex selection as self-evidently ignorant, cruel, and misogynistic – an obvious evil meriting denunciation and eradication. Drawing on ethnographic fieldwork in Gujarat state, this article zooms out from ultrasound and abortion to survey the landscape of biomedical, herbal, and religious son production techniques surrounding them. Doing so clarifies the lived moral experience in which sex selection is embedded. Resort to multiple son production techniques is both an abstract *moral indicator* reflecting prevailing concerns and a pragmatic *moral intervention* aimed at harnessing every available means in response to those concerns. Fundamentally, people live out the multimodal quest that sometimes leads to selective abortion as aspiration – social, bodily, spiritual – toward an indispensable good, not as heartless rejection of daughters. Pluralistic son production illuminates the moral uses of medical pluralism for care-seekers, social scientists, and policymakers and practitioners. The case underscores that “complementary” therapies, rather than being just desperate behaviors, barriers to biomedical therapy, or curiosities to be integrated into care, may in fact be the clearest markers of the moral conditions in which public health problems unfold.

## Introduction

Slouching on the clinic benches, Gauri-devi and Dhruv-ji awaited the illegal ultrasound that would reveal their fetus’s sex.^1^ Gauri-devi draped a *dupatta* over her head to shut out the world. Eyes closed, hands joined, head bowed, Dhruv-ji murmured prayers to Sai Baba, a nineteenth-century mystic and saint.

By then, the mild-mannered, bespectacled forty somethings – she a schoolteacher, he a bank official – had endured years of delay and discouragement. Fifteen years earlier, Gauri-devi had birthed a daughter, now their pride and joy. But stopping there had been out of the question. Almost immediately, they had started trying for a son. Facing difficulties getting pregnant, Gauri-devi had submitted to complex medication regimens, hormonal injections, even exploratory surgery. She had swallowed bitter herbal pills and powders intended to maximize the odds of a boy. Multiple times a day, every day for years, Dhruv-ji had prayed to God and Sai Baba. Three months ago, after a decade and a half of tribulations, Gauri-devi had finally conceived.

On our clandestine ride to Chetna Clinic that morning, a bump had prompted Dhruv-ji to squeeze the rickshaw driver’s shoulder: “Careful, Brother!” The “precious pregnancy,” as they called it, demanded delicate handling.

Yet everything hinged on the ultrasound. An undesired result would force Gauri-devi and Dhruv-ji to confront whether their pregnancy – so belated, so hard-won, so precious – should continue or end.

Since the 1980s, selective abortion of female fetuses has become pervasive in India. Couples like Gauri-devi and Dhruv-ji feel compelled to pursue sex determination ultrasound for reasons broadly consistent from Beijing to the Balkans (Guilmoto, 2015; John et al., 2008, 52–87; Kaur & Kapoor, 2021, 116–20; Khanna, 2009, 28–74; Unnithan-Kumar, 2011; Visaria, 2007, 154–60). Father–son connections structure descent, inheritance, and intergenerational relationships. Men generally remain with parents after marriage, while women join husbands’ families. Sons ostensibly guarantee material security and social recognition; daughters, financial and emotional liability. Given the widespread perception that sonless elders face financial precarity and social death, one boy becomes the end – temporally and motivationally – of reproduction. Amid various cultural and economic shifts, prospective parents on the path toward a son have increasingly sought to abort potential daughters as they begin accumulating; in birth data from the 2010s, the sex ratio at birth for India’ western region is essentially natural in firstborns, but falls to 0.775 girls per boy after a firstborn daughter and all the way to 0.572 after two daughters (Kulkarni, 2020, 21).^2^

Individual families’ choices have aggregated to staggering effect. Nearly 400,000 sex-selective abortions take place countrywide annually (Kulkarni, 2020, 16). In the western state of Gujarat, where Gauri-devi and Dhruv-ji’s journey unfolded, 8% of anticipated female births went “missing” in the 2010s (Kulkarni, 2020, 16). The most recent census data counted only 0.760 girls per boy in Mahesana city, where we began that morning’s trip.^3^ Despite extensive public health interventions, regional “surpluses” of unmarried men, and shifts in attitudes and practices related to gender, kinship, women’s education, and parental aspiration for daughters’ futures (Kaur et al., 2016; Kaur & Kapoor, 2021, 120–23; Kaur and Palriwala 2014), India’s trend of “missing” girls is only just beginning to turn around (Tong 2022). Each missing girl reflects an intimate quest: a couple like Gauri-devi and Dhruv-ji, often aided by a black-market broker,^4^ finds a clinician willing to perform clandestine sex determination ultrasound and – if desired – selective abortion.

Current governance interventions do not adequately address the everyday moral dimensions of this intimate quest. While abortion remains legal in India, prenatal sex determination has been criminalized since 1994. Awareness campaigns targeting sex selection, such as Gujarat’s “Save the Daughter,” have proliferated since the early 2000s. Law enforcement and public health messaging focus on condemning and extirpating selective abortion, with limited empathy for the patriarchal constraints, household motives, and reproductive difficulties that may shape pursuit of a son for people like Gauri-devi and Dhruv-ji (Eklund & Purewal, 2017; Purewal, 2014, 2018; Sandesara, in submission). Sensationalistic rhetoric about “killing” and “sin” abounds. Posters pair girls’ smiling faces with questions like “Do these eyes full of feelings and this innocent face discourage you from committing female feticide?” The overall implication is clear: “female foeticide” is self-evidently ignorant, misogynistic, and cruel, the work of either bigoted villains or hapless rubes acting out the dictates of a sexist tradition; perpetrators must be stopped, made to see the value of daughters, and induced to welcome more.

But encounters with the pluralistic care seeking of couples like Gauri-devi and Dhruv-ji has convinced me that a framing narrowly centered on the badness of biomedical sex selection misses the essence of people’s lived moral experiences. This article asks: what is the broader moral experience in which biomedical sex selection is embedded? For most people, the journey that sometimes leads to selective abortion is not a prolonged rejection of daughters, but an arduous, fundamentally positive quest for a son. While loving existing girls, families feel compelled to aspire – socially, bodily, spiritually – toward the boy that patriarchal structures render rightful and necessary. The moral dimensions of this aspiration come into clearer focus when we zoom out from the widely reviled practices of ultrasound and abortion to survey the full range of techniques for producing a son in the Mahesana region.

This article argues that medical pluralism is both a *moral intervention* and a *moral indicator* with respect to biomedical sex selection. Pragmatically, pluralistic son production is a moral *intervention* centered on harnessing every available means for securing a boy. Amid the uncertainty, desperation, and hope of son pursuit, different modalities make different instrumental contributions. People resort to multiple techniques in an attempt to grasp for pragmatic moral agency – to channel vital processes according to shared visions of the good. At the same time, more abstractly, prospective parents’ exertion through multiple complementary methods is a moral *indicator* of the positive subjective valence of a journey in which rightful aspiration predominates over evil. Encounters with biomedicine, herbal medicine, and religion all illuminate the overall experience of striving against sonlessness, which is less anti-female than pro-male-at-all-costs. For most couples, selective abortion is not an endpoint, but a waypoint in the grand moral quest for the ultimate reproductive good.

In addition to indexing the overall moral experience of son pursuit, biomedicine, herbal medicine, and religion all make specific pragmatic contributions and index specific moral concerns. Pro-conceptive biomedical treatments promise scientific control, but they cannot proactively guarantee a boy. Consequently, they illuminate how people endure chronic tribulations in pursuit of a son. Herbal regimens, by contrast, purport to proactively intervene on the body to modify virile substance. Consequently, they illuminate people’s moral anxieties about the appropriate reproduction of masculinity. Finally, religious methods also intervene proactively, but on cosmic forces. Consequently, they illuminate people’s explicit moral reflections on how personal goodness, karma, and the sin of selective abortion impact son pursuit.

Pluralistic son pursuit thus demonstrates how medical pluralism can be both a moral endeavor and a marker of moral conditions. ‘Medical pluralism’ refers to the coexistence, competition, and syncretism of diverse therapeutic systems, beliefs, and practices (Hsu, 2008; Leslie, 1980; Penkala-Gawęcka & Rajtar, 2016; Raffaetà et al., 2017).^5^ Dating back to anthropology’s colonial roots, scholars have couched non-biomedical therapies as resting on alternative “beliefs” (e.g., Evans-Pritchard, 1976; Galvin et al., 2023; Whitaker, 2003; cf. B. Good, 1994, 1–24), with the practical implication that appreciating non-scientific explanations can promote uptake of biomedicine by identifying congruence barriers, fostering cultural competency, and facilitating integrative treatment. But we can also understand pluralistic care-seeking as pragmatic improvisation amid bodily, relational, and existential uncertainty (Last, 1981; Leslie, 1980, 194; Obermeyer, 2000; Street, 2014, 46, 156, 267en23; Turner, 1967; Whyte, 1997, 24). Attention to pluralism’s pragmatic aspects can inform policy and practice, too, by highlighting lived experiences of striving. Prior scholarship on Indian sex selection has documented diverse son production techniques, including herbal treatments, special diets, torso bandaging, amulets, blessings, tantric rites, pilgrimages, and timed intercourse (Dagar, 2002, 37–46; John et al., 2008, 60–62; Khanna, 2009, 95–97). Tracing how people move through this wider ‘medical landscape’ (Hsu, 2008; see also Kołodziejska-Degórska, 2016) can illuminate pluralistic son pursuit as both moral intervention and moral indicator.

Invoking morality does not mean evaluating sex selection and its surrounding practices as good or bad; it means recognizing that they involve matters of good and bad for the people living them (cf. Laidlaw, 2014, 3). The ‘moral’ here refers not to staid rules (cf. Fassin, 2012, 9; Pandian & Ali, 2010, 2), but to pragmatic, embodied actions that dynamically address “the gap between the given and the good” (Pandian & Ali, 2010, 2).

To be clear, western Indian notions of the good take profound gender inequalities for granted. Couples like Gauri-devi and Dhruv-ji readily acknowledge sex selection and son pursuit as manifestations of patriarchal structures. But accounts of reproductive misogyny narrowly centered on selective abortion miss the problem’s lived moral complexity. While prospective parents make choices to abort potential girls, they do not do so as they please. They are constrained by social arrangements that channel necessity, entitlement, and desire into the norm of one son per family. Intervening effectively on sex selection must begin with empathically understanding the moral experience in which it is embedded.

My argument about the moral dimensions of pluralistic son production builds on over 2,000 hours of ethnographic research in Gujarat’s Mahesana District between 2012 and 2015. With the support of Uma-masi, the black-market broker who arranged Gauri-devi and Dhruv-ji’s trip, I observed 172 sex determination ultrasounds and 29 selective abortions. I spoke with dozens of clinicians and over 100 families about their experiences of biomedical sex selection. But I also observed and discussed dozens of families’ engagements with complementary biomedical, herbal, and religious practices.

Consistent with the makeup of the local population, most families I encountered were Gujarati Hindus from various caste communities. In many ways, these caste communities were their members’ functional social worlds. I did occasionally encounter Jains, Muslims, and non-Gujaratis like Gauri-devi and Dhruv-ji traveling in from neighboring states. Son pursuit took place all along the class spectrum.^6^ In my observation of biomedical sex selection and its surrounding methods, I encountered everyone from impoverished manual laborers to middle-class families like Gauri-devi’s to fabulously wealthy industrial tycoons. While attempts at son production were widely shared, such attempts meant radically different things for different families. For example, 20,000 to 30,000 rupees – the typical cost of sex determination followed by selective abortion at Chetna Clinic – was a manageable sum for the affluent, but it could eat up a month’s salary for a schoolteacher, and an entire year’s disposable income for a laboring household.

The analysis here is my attempt as a man, feminist, doctor, and anthropologist, as a U.S.-born and -based Gujarati, as a child of mixed- but privileged-caste marriage, to understand people’s moral striving on its own terms – to anthropologically confront interventions that often exceed my own understanding or credulity.^7^ It is also my attempt to witness the difficulties endured by women seeking a son within western Indian patriarchy – difficulties I cannot understand in the embodied way they do.

## *

Back at Chetna Clinic, Dr. Ranjit finally called. Inside, Dhruv-ji, now trembling, stared at a photograph of Sai Baba. Gauri-devi’s jaw tightened, and her eyes closed.

After hours of anticipation, the scan lasted ten minutes. Once Dhruv-ji produced payment, the doctor nodded gently.

“It’s all set. Male.”

Gauri-devi and Dhruv-ji mouthed the last word in disbelief. After Dr. Ranjit repeated it twice, the couple erupted in celebration. Dhruv-ji vowed to forever keep the doctor in his prayers “for this very good thing you’ve done for us.” Gauri-devi sobbed convulsively. For several minutes, the doctor and I witnessed an outpouring of joy, jubilation, relief, and tenderness different from everything in caricatures of wicked, daughter-hating parents. Gauri-devi and Dhruv-ji did not have to consider ending their precious pregnancy. They had not yet reached the end of reproduction, but that scan was a kind of culmination in their struggle against sonlessness.

Wiping her eyes and exiting into the sunshine, Gauri-devi said, “I used to feel so sad. We’re just two sisters. My father was murdered when I was young. And I’ve always felt it, that I have no father, no brother, no son. So, I always felt that if I got a boy in my home, I would feel…” She trailed off. The visit had fulfilled not just fourteen years, but a lifetime of aspiration.

Couples like Gauri-devi and Dhruv-ji endured prolonged, ambivalent ordeals of “reproductive chronicity” (Lukšaitė, 2022, especially 313, 314, 319-320, 322; see also Majumdar, 2023) – of conjoined suffering and care – to secure the boy that patriarchy rendered rightful and obligatory. Son pursuit involved a coin toss, a fifty-fifty proposition subject to bodily vicissitudes and fate. While care-seeking is often fundamentally open-ended (B. J. Good et al., 1994; Mattingly, 1994; 2010; Samuels, 2018), son production treatments had a clear endpoint: the necessary boy. Much like the ‘quest for conception’ (Inhorn, 1994; cf. Bharadwaj, 2016, 24–88, 214–42), the quest for a son entailed striving according to a culturally defined plot. Hope charted a straightforward path: conception, ultrasound, birth. Biomedicine, herbal medicine, and religion all became technologies of hope (Franklin, 1997), enabling “embodied investments in specific cultural values” (Franklin, 2006, 549). But one paradox of hope is that it is always “poised for disappointment”; it “asks for more than life promises” (Mattingly 2010, 3; cf. Franklin, 1997, 193, 224en20). The normative path could fold back on itself in spirals of frustration when infertility, miscarriage, female births, or selective abortion derailed progress. Nonetheless, people continued framing such derailments as setbacks within an overarching positive journey out of sonlessness.

Below, I review how couples like Gauri-devi and Dhruv-ji engaged with pro-conceptive biomedicine, herbal medicine, and religion as modalities of son pursuit. The article’s conclusion returns to the moral uses of medical pluralism in social life, social analysis, and social praxis.

### Biomedical Techniques: Reactive Agency and Reproductive Endurance

In Mahesana District, where pregnancy had long been medicalized and commercialized (De Costa et al., 2014, 3; Government of Gujarat, 2009; IIPS, 2010, 12, 64, 67), much of people’s striving unfolded through an obstetric trial and error that could span chronic fertility treatment, antenatal care, pregnancy loss, and recurrent female births alongside selective abortion. While families valued biomedicine as a modern, scientific technology of hope, its locally available techniques afforded limited control over ‘selective reproduction’ (Wahlberg & Gammeltoft, 2018). Pro-conceptive treatments produced and maintained pregnancies without increasing the likelihood of a boy; ultrasound enabled mere reaction to an already-formed fetus. Consequently, biomedical son pursuit entailed repeated coin tosses, and experiences of it illuminated the moral dimensions of enduring for a boy.

As our rickshaw puttered back from Chetna Clinic, Gauri-devi and Dhruv-ji, flush with relief, opened up about their lives, their careers, and their daughter, a stellar high school student. They also narrated their laborious quest for a son.

Very early in their marriage, unprepared for children, they opted for abortion – a decision they later rued. Gauri-devi subsequently conceived, gestated, and delivered their daughter. Then, the trouble began. After “waiting, waiting, waiting for almost ten years,” attempting conception without treatment, they sought local fertility care in Rajasthan state. Four fruitless years later, they turned to Gujarat, since “people say the technology’s a bit better here.” Every fortnight for eight months, they took an overnight bus to Mahesana, spent all day there for a five-minute clinic visit, and returned home overnight. The treatment included more pills, injections, and procedures than Gauri-devi could remember.

“For us,” Dhruv-ji sighed, “just the pregnancy’s a big deal. To me, it’s a miracle it happened naturally! I thought we’d have to go for a test-tube baby.”

Gauri-devi recalled hearing how other women underwent multiple selective abortions, leaving their bodies “ruined” or “finished.”

“You must understand that as your penance,” Dhruv-ji offered eagerly. “So many people have seven-eight. Imagine how troubled they feel! But penance brings a result. Whatever happens in the middle, that’s well and good.”

Gauri-devi harrumphed. She had accepted great suffering, but it had been more than a temporary nuisance. She knew, in an embodied way, how women endured biomedical interventions without any guarantee of a son.

To be sure, biomedical technologies for proactive son production had long existed (cf. Allahbadia, 2002; van Balen & Inhorn, 2003), but few Mahesana-area families enjoyed access. While local obstetricians were familiar with techniques for producing Y chromosome-enriched semen samples or genetically testing *in vitro* fertilization (IVF) embryos, legal restrictions made it virtually impossible to commercialize these. Doctors did describe very affluent patients going abroad to become ‘reproductive outlaws’ (Inhorn, 2015, 175); one explained, “In Thailand-Dubai, selective transfer is perfectly legal. Couples go, get IVF, get pre-diagnosis and selective transfer, and come back home with a designer pregnancy.” But such examples were exceptional. Illegality and cost made proactive biomedical intervention unattainable for most.

Consequently, striving biomedically for a son entailed laborious ordeals that conjoined suffering and care. The story of Vinita-ben, an impoverished blacksmith’s wife, was typical. I met her at a routine prenatal visit. She and her husband had two daughters, four and seven. After the second, they had undergone twenty-four months of costly fertility treatment with two separate doctors. They had almost given up when a “miraculous pregnancy” ensued. But they had aborted when ultrasound revealed “yet another girl.” Two subsequent pregnancies, both scanned as “male,” had ended in miscarriage. Facing poverty, advancing age, and the wear of repeated gestation, the couple felt desperate for a son.

Vinita-ben’s experience, like Gauri-devi’s, exemplified the burdens women bore amid the reproductive chronicity of son pursuit. Many felt physically and emotionally spent after years of fertility and antenatal care. Many lamented costs. A barber’s wife once told me, “We needed treatment for four years! Do you know how many shaves it takes to gather 80,000 rupees? After all that, to have a girl? Of course we were disappointed.”

As this comment suggests, unfavorable sex determination results could derail quests for a son even after significant investment. Following a third miscarriage, Vinita-ben and her husband conceived again, only to receive another “female” result; they opted for abortion and decided to stop trying. They explained the decision to end a long-awaited pregnancy, amid tremendous disappointment, with the same simple declaration many couples offered: “All this effort was for a boy.”

Women like Vinita-ben and Gauri-devi viewed selective abortion unfavorably, but they were willing to contemplate it in service of securing a son without accumulating daughters. Sex-selective termination, with its second-trimester timing, was widely considered physically arduous. It was also considered sinful, since it ended a more developed potential life – one already recognizable as a specific kind of future relative. Rejecting daughters *qua* daughters did feel wrong to most couples. But it was a distasteful means toward a rightful end.

In extreme cases, “female” ultrasound results prompted families to end pregnancies conceived via expensive, burdensome, hopeful IVF treatments. Such cases underscored people’s experience of sex selection not as an evil freely chosen, but as a misfortune suffered. They laid bare the positive aspiration lighting the path to selective abortion, along with the pragmatic limitations of biomedical son production.

I once observed Dr. Ranjit scan a forty-something patient carrying twins. After twelve years of infertility care, she had finally conceived via IVF. While scanning, the doctor asked, “If you’ve gotten pregnant after twelve years, what’s there to see? Keep it, no?”

“But we already have a girl, right?” the patient replied curtly.

Eventually, the doctor sighed, “Both are female.”

Out in the waiting room, the patient informed her husband. He shook his head.

“We want boys,” he told me, thumping his chest. “Or a boy and a girl…. We came here to get the boy-girl thing done, but inside, I’m sure I have two boys! Maybe he can’t see it exactly.”

When Dr. Ranjit returned from lunch, the husband marched in.

“She says you said it was female,” he rumbled. “That’s not possible. I’m sure I have two males! Please look again.”

Grudgingly, the doctor agreed. As he scanned, the husband thundered, “We already have a girl! We got pregnant after so many years, so it must be a boy! We must have two boys. Look at it!”

The doctor attempted to demonstrate on-screen anatomy, but the husband interrupted: “I, Saheb, have a boy! 100%, I don’t have a girl! You can’t confuse my missus …. She’s so tense she barely talks at home. Saheb, you tell her, or she’ll be left with tension. Even if it’s one boy, one girl, I want to keep it.”^8^

“Think of it that way,” Dr. Ranjit suggested, smiling uncomfortably. “’It’s one boy, one girl. The doctor’s wrong, we’re right.’”

“Why don’t you say it once?” the husband insisted. “*I’m* certain. But if you say it once, then my wife’ll be satisfied. Say it once, Saheb, that it’s a boy.”

Dr. Ranjit blandly offered to re-scan a week later. After an awkward silence, the man urged his wife, “Now, don’t take any tension: ‘Oh, it’s two girls.’ It’s *that*. We’ll come back, sure, but banish the tension. It’s over!” The patient frowned. Mumbling farewells, the couple left.

The following week, a broker promising a second opinion inadvertently brought them back to Chetna. On re-scan, the result was unchanged.

Months later, Dr. Ranjit casually mentioned, “Oh, you remember that couple with IVF twins? They came back. They finally got termination here, at four months!”

After winning the battle against infertility, the couple had lost the coin toss. The husband’s insistence, the search for second opinions, and the one-month delay between ultrasound and abortion all reflected the difficulty of accepting that a rightful quest had gone unfulfilled. The woman’s abortion following IVF made sense, because pro-conceptive biomedicine had failed to produce the boy she so desperately sought. It was an instance in which the very “care practices… employed to manage… reproductive lives… emerged as ambivalent acts that merged with reproductive suffering” (Lukšaitė, 2022, 314).

I also witnessed a rare case where the prospect of proactive biomedical intervention briefly tantalized, only for legal restrictions to snatch it away. The couple came to Chetna from hundreds of kilometers away, toting the youngest of three daughters. Recent fertility treatment had produced another three fetuses. Because triplet gestation posed significant risks, their regular obstetrician had recommended that a specialist perform ‘selective reduction’ – *in utero* abortion of one fetus. Given the rare chance to shift odds biomedically, they were seeking guidance.

After scanning, Dr. Ranjit explained, “You have two females, one male.” Clutching the toddler tight, the parents nodded. “But when you do the reduction, you can’t say you want a female reduced.”

Husband and wife froze.

“They’ll probably do a female,” Dr. Ranjit continued, “because they go for the most accessible one. A female’s in front. The male’s lower. But if they accidentally inject the male, then everything’ll be ruined! You’ll have to come back for confirmation. Remember, you can’t say anything to them!”

“Can’t we suggest?” the husband asked. “Or could *you* tell them?”

“No! I can’t say anything, can I?”

The husband muttered, “Okay, suppose the male gets reduced—”

“Then it’ll be a big problem!” Dr. Ranjit hissed. “Then you’d have to get rid of the whole thing, right?”

The couple looked down and nodded slowly.

“That probably won’t happen,” the doctor continued, composing himself. “They’ll do the front one.”

The husband persisted, grasping for words: “So could we meet him—ask him—and then, maybe if we don’t do it—would that be okay? He’ll tell us which one he’s going to remove, right?”

“Brother, you can’t ask! He’s just going to say, whichever one comes first—”

“And that one’s female?”

“Yes, that one’s female. This is just, by chance, if something happens. You’ll have to come back once. No charge then.”

After paying a hefty fee, the couple issued one more plea. The doctor refused. They bowed meekly, gathered up the toddler, and left.

Weeks later, I asked Dr. Ranjit what had happened. He shook his head and sighed. The specialist, he told me, had injected the male fetus, leaving two female fetuses. The couple had opted for abortion.

“This is called hard luck, eh?...” the doctor said. “They already had three females, after all. What would they do with two more? All their effort ended up wasted, didn’t it?”

The case was unusual: ultrasound produced knowledge that could have enabled proactive intervention for a boy. But the knowledge’s illegality left it painfully unusable. Ultrasound ultimately ended up playing its usual role: revealing the results of a chance process. As in other cases, a pregnancy produced and gestated with great hope suddenly became unwanted.

Abstractly, the tremendous money, energy, time, and aspiration that sonless families poured into pro-conceptive biomedicine reflected moral conditions that rendered a son the ultimate reproductive good. Pragmatically, though, biomedical son pursuit was a series of coin tosses. Mahesana-area obstetric care fostered sex-nonspecific pregnancies, then revealed already-formed fetal sex. Proactive moral intervention on the high-stakes coin toss of son production required other modalities.

### Herbal Techniques: Proactive Son Production and Managing Masculinity

Families’ resort to herbal treatments indexed a yearning for more active control over the grand moral quest for a son. India has long boasted various herbal methods for increasing the chances of a boy (Bandyopadhyay & Singh, 2007; Dagar, 2002, 23, 37–38, 44; Jeffery et al., 1989, 76, 191–93; John et al., 2008, 60, 61–62; Neogi, 2021; Stork, 1992, 92–93).^9^ Around Mahesana, such methods divided into two types. One operated through pregnant women, putatively boosting the developing embryo’s masculine substance. The other consisted of preconception pills for men that supposedly increased Y chromosome-bearing sperm. Both types responded to anxieties about diminishing virility, reflecting the moral weight western Indian patriarchy imposed on the appropriate propagation of ‘lineal masculinity’ (King & Stone, 2010). Pragmatically, both types were one degree removed from biomedicine – still corporeal in their targets and ostensibly scientific in their mechanisms, but more proactive in producing a boy.

As our rickshaw clattered on, Gauri-devi and Dhruv-ji narrated the son production treatments they had obtained from two Ayurvedic practitioners located hundreds of kilometers apart. Shortly after conceiving, Gauri-devi had received several injections. She had also started a strict monthlong oral regimen involving multiple daily administrations of powders, tablets, and liquids. Both therapies exerted “an effect on hormones,” Dhruv-ji explained, directing “the embryo’s development toward the male sex.”

Knowing Dhruv-ji loved biology, I asked how the treatment worked if sex was fixed at conception. He shook his head:

They say that, but I don’t find the science so firm. There are so many cases with this medication, and they’re all male! How, if the sex is decided? Some modification must be possible early on. How does science know it’s this or that in the beginning? And there’s nothing developed in science that allows you to separate X and Y to get just one.... So, this is another method. It’s hard to say what the success rate is.

“If you do it and get a result,” he shrugged, “it’s true.”

Certainly, I thought to myself, the first-trimester timing and Dhruv-ji’s explanation might look different with access to sperm sorting or early genetic testing. Moreover, the known and unknown burdens of the mysterious regimen – punctilious compliance, culpability for failure, bodily complications – all fell on women like Gauri-devi (cf. Bandyopadhyay & Singh, 2007).

But I was partly missing the point. I was not considering how Gauri-devi and Dhruv-ji experienced herbal treatments as parts of a broader aspiration toward a culturally normative good – as technologies of hope directed toward the rightful end of reproduction. Herbal son production promised otherwise-elusive bodily control while addressing moral anxieties about son necessity, household reproduction, and masculinity amid shifting social conditions – anxieties that fit into a pattern in Indian patriarchy whereby gender and personhood come to constitute one another (Busby 2000; Lamb, 1997, 2018). I encountered widespread concern about how “men today are no longer men” – about how “plastic” environments, modern diets, vices, and sedentary lifestyles were diminishing sperm counts, virility, and the ability to father children, particularly boys. People cited press reports on the supposedly widespread sperm count crisis, but they also invoked gender ideologies, long enshrined in Hindu scripture, ritual, and lay discourse, that linked together masculine personhood, bodily substance, and capacity for procreation (Busby, 1997; Dube, 1986). *Ultimately*, many people told me, *this is all a men’s problem. After all, women are just the field, right? Men provide the seed. If that seed is not good, how can the children be good?*

Even when administered during the first trimester to women like Gauri-devi, herbal techniques promised to rectify questionable virility and proactively produce sons. This was evident in the practice of an uncredentialed Ayurvedic healer who styled himself Shastri-ji – the Learned One.

Upon meeting me, Shastri-ji declared, “I’ve gotten boys for 4,000 families!” The causative verb he used – *apaavvu* – is difficult to translate. Narrowly meaning “to cause to be given,” it applies to actions like securing something for another through imposition on a third party. People used it to describe the action of herbal healers, religious ritualists, and obstetricians in producing a son, the implication being that the practitioner had effected a bestowal from nature or God.

Shastri-ji administered a seven-day, home-compounded treatment with characteristics similar to Gauri-devi’s oral regimen: mandatory first-trimester timing, fastidious requirements, and purported augmentation of embryonic masculinity. Treatment needs had increased in recent years, Shastri-ji said, since various poisonous substances – tobacco, alcohol, spicy and fried food, processed food, South Indian food – had led to “decreased masculine essence in the body,” “impotence,” and “sperm counts of zero.” He assured me the regimen almost always produced a healthy boy while acknowledging the impossibility of “making something out of nothing” when husbands had “zero sperm.” With its emphasis on mitigating insufficient paternal virility, Shastri-ji’s regimen treated not just bodies, but also moral anxieties and hopes about individual, household, and social reproduction.

Many families, skeptical about changing sex after fertilization, instead sought out preconception herbal regimens targeting prospective fathers’ “Y count”; just as clearly as first-trimester treatments, these regimens reflected prevailing concerns about both proactive control and diminished masculinity. Dr. Rajen, a credentialed Ayurvedic doctor, prescribed industrially manufactured herbal “male enhancement” pills with names like Y-Spur, Y-Grow, and Alto-M. Marketing materials claimed the pills fixed poor libido, erectile dysfunction, low sperm count, and “seminal debility” while facilitating “deep penetration” and “impregnation.” According to Dr. Rajen, they also tilted the coin toss by increasing “Y sperm.”

Dr. Rajen framed his regimen as a bioscientifically logical intervention for securing the necessary son while avoiding selective abortion. Because Dr. Rajen brought current clients to Chetna for black-market ultrasounds, I saw how he opportunistically pitched this framing to post-abortion families on the clinic’s wards: “Say, how many girls before this?... Well, before the next pregnancy, come see me for Ayurvedic treatment. You’ll get a result, 1,000%! See, you must start treatment for the man three months beforehand. Better than all this headache!”

Outside the sales pitch, Dr. Rajen admitted his method was probabilistic, not deterministic: “You get sixty-seventy percent success. Even those who keep having girls, many have no idea there’s a treatment for this! They just keep getting pregnant, checking at three months, and getting it taken out after a negative report. So, for them, we’re increasing the chances…. If they have a negative report, I give them back their money.”

Dr. Ranjit privately scoffed that Dr. Rajen could return payments because he sold his regimen at an astronomical markup, repackaging 100 rupees worth of pills and charging 10,000. The obstetrician scorned the Ayurvedic practitioner as “a quack only,” alleging that more than half the patients he brought for ultrasound ended up having female fetuses. Dr. Ranjit’s obstetric colleagues widely shared the skepticism about herbal healers.

More broadly, obstetric, herbal, and religious practitioners all expressed skepticism about each other’s son production methods, reflecting how medical pluralism often engenders contention over biomedicine’s supposed hegemony (Baer, 2001; Halliburton, 2023; Hollenberg & Muzzin, 2010; Lambert, 2012) and strategic claims to separation of systems (Cremers, 2019; Eves & Kelly-Hanku, 2020; Hampshire & Owusu, 2013; Pinto, 2015). Dr. Rajen dismissed post-conception herbal treatments as scientifically nonsensical. Shastri-ji faulted credentialed Ayurvedic practitioners for using mass-produced pills. Both, together with ritualists, observed that obstetricians were powerless to alter fetal sex. Obstetricians, conversely, frequently derided herbal and religious practitioners as unscientific and unproven. Obstetricians and herbal healers condemned ritualists as merchants of “blind faith,” while ritualists insisted no bodily intervention would fix cosmic problems.

Yet biomedical, herbal, and religious son production all made pragmatic contributions to the same overarching moral quest, with areas of overlap exemplifying how medical pluralism can involve strategic boundary crossing as well as competition (Lang & Halliburton, 2023; Langford, 1999; Zhang, 2007). Biomedical and herbal practitioners recommended prayer alongside medical treatment, casting bestowal of children as ultimately dependent on karma, fate, and divine providence. Herbal and religious practitioners frequently encouraged patients to pursue sex determination ultrasound to confirm treatment efficacy. (Various mitigating factors, from inadequate paternal virility to opaque karma, ensured “female” results did not challenge overall credibility.) The focus on chromosomes and embryonic development in explanations for herbal treatments like Shastri-ji’s and Dr. Rajen’s appealed to educated couples like Gauri-devi and Dhruv-ji, for it set up an intervention simultaneously comprehensible through biomedical knowledge and impossible through biomedical practice; it “borrowed” biomedicine’s legitimacy and then repackaged it into something novel (cf. Pinto, 2008, 106–40; 2015; Unnithan-Kumar, 2004). Tellingly, when families grappling with the medical, emotional, and moral aftermath of selective abortion asked Dr. Ranjit and other obstetricians to suggest medications “for next time, so this doesn’t happen again,” they often shrugged and wrote out regimens like Dr. Rajen’s.

Obstetricians’ recommendation of herbal treatments despite reservations underlined how those treatments, biologically plausible or not, allowed people to grasp for the rightful, indispensable boy. Herbal treatments made a pragmatically distinct contribution to moral quests. They reshaped and rechanneled masculinity. They held out the allure of moving in a positive direction from jump, ideally bypassing selective abortion altogether. They offered to rig the all-important coin toss. Nonetheless, if ultimate reproductive causality extended beyond the physical, to the metaphysical, yet another modality of son production might be needed.

### Religious Techniques: Cosmic Control and Moral Confrontations

Many families striving for a son turned to religious techniques alongside or instead of biomedicine and herbal medicine. Families spoke of the need for *dava* and *dua* – pills and prayer, or more broadly, medicine and religion. When reproductive misfortune, struggle, and hope exceeded medicine’s explanatory limits, ‘clinical theodicies’ (Bharadwaj, 2006) filled the void. Deities became cohabitant with humans, engaging people through diverse relations of contract and coercion (cf. Fiks, 2023; Flueckiger, 2017; Singh, 2015, 38–58; Roberts, 2012; 2016). Shrines, rituals, and personal relationships with God turned into sites of assisted reproduction (cf. Goslinga, 2011). Families pursuing a son resorted to two types of religious techniques: personal bargains and ritual contracts with the divine. Both brought people into confrontation not only with the general moral concerns of son necessity, but also with fundamental questions about personhood, karma, and merit. Pragmatically, religious techniques were two degrees removed from biomedicine: like herbal medicine, they operated proactively rather than reactively, but they went beyond bodies to address cosmic forces.

During our rickshaw ride back from Chetna, Dhruv-ji described praying to God and Sai Baba multiple times a day, every day, for years. He continued:

I told her this! ‘God won’t give you the chance to commit *paap* [sin].’ We didn’t think about abortion at all. What need was there? God was bound to do good. God didn’t even give us the occasion to think about all this: *Keep it, or not? How to care for a daughter at the age of forty? Get an abortion, or not?*....

God only does good. He won’t let bad things befall a person. I had faith from the very beginning. Why would God have us go through such a long process, just to have it taken out?... Fifteen years, we tried, tried, tried without a result. That’s punishment! Well, God wouldn’t give us another punishment after that…. God can’t do such a great injustice, piling another injustice on top of fifteen years of injustice.

“God wouldn’t do that to good people,” he finished simply.

Like Dhruv-ji, many people felt cosmic forces helped determine whether and how they attained the rightful son. They invoked karma, *nasib* [fortune], kismet, *kudrat* [nature], God, various deities, and “what is written,” typically tying them together into a unitary whole. Families framed infertility, the trying wait for sex determination, and female results as God-given penances. They also framed the eventual son as a God-given inevitability – the fruit of good deeds, good character, prayer, and faith.

People assumed, as Dhruv-ji did, that God understood patriarchal structures and patriarchal reproduction. Given prevailing kinship arrangements, people knew God would see a female fetus as a setback on the rightful journey to completing a family – a “punishment” or “injustice,” as Dhruv-ji said. Experiences of gendered hope and disappointment transformed into matters of divine will: *God isn’t seeing that it’s our turn, he just keeps giving girls… After this girl, God’ll definitely give a boy!*

As people understood it, the divine family planning process accounted for past deeds and moral deservingness, such that coin toss results were verdicts on parental goodness. Some examples were spectacular. One woman recalled how an acquaintance had drunkenly deprecated a mother goddess’s powers after ultrasound confirmed a male fetus, only to receive a girl at delivery: “Mata-ji switched the child!” Other examples were more ordinary but no less powerful. Prospectively, men took potential sons as motives for moral improvement, pledging to forgo longstanding vices – tobacco, alcohol, gambling – if the universe favored them. Retrospectively, “male” results confirmed virtue for couples like Dhruv-ji and Gauri-devi. Those receiving “female” results, by contrast, interrogated character flaws and past mistakes, as one man did in my presence while his wife underwent selective abortion: “We requested God to hear our prayer, understand our situation, give us something good. In the end, it must be our fault. Will we fault God?... It must be something in our karma; otherwise, we’d expect God to do good for us. It’s not as if we like *this* misdeed [of abortion], either. But then, how many girls can you gather?”

Like this man, the mostly Hindu families I encountered generally viewed abortions – including sex-selective abortions – as sinful, but on a relative scale. The procedures were not absolute violations; they were proportional transgressions within a broader karmic balance of *paap* and *punya*, or meritorious deeds. Early abortions, undertaken on a tiny conceptus without sex-selective intent, were minor sins. Sex-selective abortions, which eliminated a better developed fetus, already recognizable as a daughter-to-be, were somewhat worse. But circumstances could still justify the latter. Sex-selective abortion could be undertaken as a pragmatic moral action – a “necessary sin,” as many put it, echoing conceptions well documented in moral worlds as distinct as Southeast Asian Buddhism (Whittaker, 2004, 109, 133–34; see also Gammeltoft, 2002) and Greek Orthodoxy (Paxson, 2006). Religious exhortations of the sort Dhruv-ji made to Sai Baba were partly about appealing for help in avoiding what would otherwise feel like an unavoidable sin.

When people bargained through personal prayer, as Dhruv-ji had done, they were frequently trying to escape a spiral of divinely ordained setbacks. In many such cases, like Mina-ben and Gajendra-bhai’s, the evil of sex selection was a starting point for negotiating a path out of reproductive chronicity and toward the rightful son.

When Uma-masi and I accompanied Mina-ben and Gajendra-bhai to Chetna, they had two girls in tow – a preteen and an infant. In the previous pregnancy, an Uma-masi-brokered scan had revealed a future daughter, but the couple had decided to welcome her. This time, the result at Chetna was “good.”

“What did I tell you?” Uma-masi cried gleefully. “With her, I told you, ‘Keep it. You don’t want *paap*. If it’s in her destiny, she’ll bring a brother.’ And she brought one!”

A month later, I visited Mina-ben and Gajendra-bhai’s village. There, they narrated a taxing reproductive journey.

Their first child, a boy, was born with a cleft palate. Despite costly medical treatments, he died in infancy. A year later, Mina-ben delivered the elder daughter, now almost twelve. When they started trying again for a son, four consecutive pregnancies ended in miscarriages. Their infant daughter had finally arrived a year ago. In a few months, the long-anticipated boy would hopefully follow.

Reflecting on the death of their firstborn and the string of miscarriages, Mina-ben shook her head: “God made it like that. We argued a lot with God in our grief! But it was written in our karma, in our fortune. These losses happened because of whatever was written in our karma. If we are true,… the universe will eventually look toward us, no? We have to assume that we committed *paap* at some point, and that it got in the way.”

She explained that the couple had struck a very specific bargain with God after the prior sex determination ultrasound: “We told Him to look out – ‘We’ll bring her home, raise her. But then You look out for us!’ Uma-ben suggested it, and it was our will, too. We kept thinking, ‘What if it’s in her fate to have a brother? If we bring home another girl, if we don’t do foeticide, maybe God’ll give us a boy.’”

“Honestly,” Gajendra-bhai chimed in, “we weren’t thinking of keeping it. But then we came home and decided to welcome whatever it was, to not do wrong. We thought, let’s not kill it. Because it’s foeticide…. We thought, ‘This is nature, and you can’t change nature. If we do foeticide and get pregnant after six months, it’ll be the same thing.’”

“If we’d gotten it done,” Mina-ben said, “God wouldn’t have favored us. The *paap* would’ve gotten in our way… We’d keep getting girls, again and again, because we didn’t accept our fate. But we accepted it! We accepted her, and it was in her fortune!... We figured God would give us a son. We petitioned the universe every day: ‘Look out for us, now that you’ve given two already!’”

For Mina-ben and Gajendra-bhai, a personal bargain with God, rather than selective abortion, made sense as the next pragmatic step in the quest for a boy. But that bargain had become fully meaningful only because of sex determination ultrasound, which facilitated recognition and acceptance of the potential daughter as such.

Despite their professed faith that the universe wouldn’t do them wrong, the couple had experienced intense anxiety during the recent Chetna visit.

“When Uma-masi told me,” Mina-ben recalled, “it was a relief. Honestly, this was the universe’s work, God’s. Otherwise, if it was a girl, what would we have done?

She arched an eyebrow.

“Cancel. Definitely. No doubt. That’s it.”

She shrugged.

“What else could we do? We have two already!”

If the universe had not fulfilled the agreement, the couple would have felt compelled to sin.

When I returned to Mahesana, Uma-masi added a new dimension to the bargain: “I’ve done so much for them, saved them a lot of money. I took them to Dr. Vinay four times for abortion. I got it looked at for less, finished off for less. Finally, with this baby girl, I told them, ‘Keep it.’ And now, this boy!”

Suddenly, the “miscarriages,” the arduous wait, and the bargain with God congealed into a different picture. It was a picture Mina-ben and Gajendra-ben eventually filled out for me themselves. Mina-ben’s scenario of fate sending endless female fetuses to force a second daughter was no hypothetical. It was the lived ordeal from which their bargain offered an exit. They agreed to stop sinning, provided the universe fulfilled their quest by delivering the rightful son.

Unlike Mina-ben and Gajendra-bhai, many couples opted for more formalized divine contracts. A *baadha* was a ritual agreement, usually with a Mata-ji (mother goddess), pledging an offering in exchange for a desired object (cf. Fiks, 2023, 1–4, 9–13; Flueckiger, 2017). Religious mediums known as *bhuvas* (see Prasad, 2007) performed esoteric rites to communicate with the deity, who transmitted requests onward to the “writer of fates.” A senior *bhuva* once told me that while every young woman’s fate had a boy written in it, various occult impediments - black arts, negative karma, poor fortune - trapped some in sonlessness; a *baadha* initiated before or after conception could overcome such mysterious forces.

Sometimes, people used *baadhas* and biomedical sex selection simultaneously. Gita-masi’s son started drinking excessively after his second daughter’s birth. Gita-masi and her daughter-in-law undertook a *baadha* pledging his sobriety in exchange for a boy. When the young woman became pregnant, the Mata-ji, through her *bhuva*, gave permission for sex determination ultrasound. The test revealed the desired result. Shortly after the boy’s birth, Gita-masi’s family traveled to the Mata-ji’s chief pilgrimage site, where they offered cash, coconuts, sweets, and various other sacrifices at the cost of over a month’s household income.

*Baadhas* and ultrasound could also substitute for one another. Gita-masi’s neighbor’s daughter, frustrated after three consecutive sex-selective abortions, undertook a *baadha* for a boy. The *bhuva* stipulated that sex determination would be a betrayal - a sign of “false faith” so disappointing that the goddess might turn a male fetus female. As a result, the *baadha* replaced biomedical sex selection. Conversely, many couples pursued biomedical sex selection after exclusive *baadhas* in previous pregnancies failed to bear fruit. Such couples did not necessarily forgo religious bargains completely, but they became unwilling to wait until birth to learn the coin toss’s outcome. If ultrasound suggested a failed *baadha*, they would contemplate the “necessary sin.”

Simultaneity or substitution was less about competing explanations than about pragmatic striving toward a desperately desired good. People agreed that both divine and profane forces – both fate and chromosomes – mattered. Metaphysical and medical interventions offered different ways of grasping for a son while avoiding a daughter, and people resorted to one, the other, or both according to their inclinations, circumstances, and histories. *Bhuvas* insisted they were merely “gateways,” “advocates,” “mediums,” or “proximate causes,” transmitting requests related to a process where success ultimately depended on faith, karma, and a cosmic agency whose workings exceeded comprehension. Similarly, people did not necessarily engage with *baadhas* credulously: ritual agreement was one among multiple technologies of hope that families might concurrently use in their high-stakes quest.

*Baadhas* and prayer both reflected people reaching for proactive control that went beyond the body, to cosmic forces. Prospective parents offered up goods – coconuts, cash, devotion, virtuous behavior - and evils – vices, wicked behavior, sex-selective abortions – in exchange for the ultimate reproductive good. Because they involved explicit deliberation on good and bad, religious son production methods provided a particularly clear window on the moral experience of aspiring within the confines of patriarchy.

### Conclusion: The Moral Uses of Medical Pluralism

The widely reviled practices of sex determination ultrasound and selective abortion come into rather different focus when we zoom out to take in the complementary son production methods surrounding them. The overarching moral experience of son pursuit becomes clearer. In the Mahesana region, couples struggling against sonlessness turn to fertility treatments and intensive antenatal care, Y-boosting pills and embryo-molding packets, personal prayers and divine contracts as different techniques for channeling vital processes toward the ultimate reproductive end. People’s hope, desperation, and endurance in pluralistic son pursuit reflects both the astronomically high stakes of having a boy and the ways lived experience configures the journey that sometimes leads to selective abortion as one of positive aspiration.

Pragmatically, pluralistic son pursuit is a *moral intervention* aimed at harnessing every available resource – bioscientific and herbal and religious, physical and metaphysical, reactive and proactive – for pursuing a boy. Biomedicine’s fertility treatments, antenatal regimens, and ultrasound scans afford limited, rather reactive control over son production. Herbal regimens promising alteration of “Y count” or embryonic substance offer more proactive control, but they remain tethered to the body. Religious bargains transcend the body, proactively intervening on cosmic forces. In navigating a vast medical landscape of biomedical, herbal, and religious techniques, people improvise pragmatic agency amid high stakes and uncertainty.

More abstractly, pluralistic son production is a *moral indicator* that illuminates the positive valance of the broader experience in which sex determination ultrasound and selective abortion are embedded. The totality of people’s exertion shows how, subjectively speaking, normative aspiration predominates over the badness of sex selection. While the labels families apply to female sex determination results – “bad,” “negative,” “unjust” – are obviously negative evaluations of girls, people understand them primarily as lamentations about the elusiveness of an indispensable social good. Even though the quest for a son can be a long and arduous journey, full of uncomfortable confrontations with masculinity, littered with the traps set by past karma and the prospect of selective abortion, the quest is ultimately a rightful journey. When people reject potential daughters, they experience the act primarily as a setback on this journey – a misfortune produced by uncontrollable social, bodily, and cosmic forces, not a misdeed freely chosen.

Prospective parents’ suffering and endurance in the pluralistic journey of son production are testaments to the force of patriarchy’s compulsion. Social arrangements channel necessity, entitlement, and desire into the norm of one son per family. People do not mindlessly act out this norm as automata, as mere instruments of social structure. They live it out in thought, feeling, and action – pragmatically, poignantly, reflectively. The experiences of couples like Gauri-devi and Dhruv-ji drive home how, in son pursuit, it becomes difficult to neatly separate good and bad, nefarious and non-nefarious, hopeful and aversive. Pluralistic son production exemplifies why “an anthropology of the good” focused on understanding and pursuit of moral goods (Robbins, 2013) is not so easily separable from a “dark anthropology” focused on oppression and suffering (Ortner, 2016).

This article’s analysis has implications for the moral uses of medical pluralism among both care-seekers and social scientists. Attention to pluralism can clarify what matters, pragmatically and existentially, in social problems involving biomedicine and other healing systems. Illness, misfortune, and care are windows on the moral dimensions of social life (Kleinman, 1988; Ong, 1988; Petryna, 2002; Turner, 1967). Pragmatic, improvisational engagement with multiple care modalities can manage moral and social relations alongside bodies (Brodwin, 2003; Livingston, 2012; Street, 2014; Unnithan-Kumar, 2010; Whyte, 1997). Pluralistic son production demonstrates how the clearest insights on the moral meaning of a practice like sex selection may come not from drilling into the practice itself, but from widening to survey the landscape around it.

This article’s analysis also has implications for policy and practice around medical pluralism. Complementary therapies are not just desperate behaviors, barriers to rational therapy, or curiosities to be integrated into and superseded by biomedical management. They may be the clearest markers of the moral conditions in which public health problems unfold, pointing the way toward more realistic, community-responsive governance. Complementary son production therapies, for instance, suggest the incongruence and logical flaws of institutional interventions centered on narrow views of ultrasound and abortion. Crafting more effective responses to sex selection must begin with more empathically understanding people’s subjective moral experiences of it within patriarchal social structures.

## Data Availability

Because the research data on which this article is based contain copious references to criminal activity, they cannot ethically be deposited in a public repository.

## Footnotes

1 All personal names except Sai Baba’s are pseudonyms, as is “Chetna Clinic.” Matching my address of research participants, I add the title ‘Dr.’ and the honorific suffixes ‘-ji,’ ‘-devi’ [goddess], ‘-ben’ [sister], and ‘-bhai’ [brother]) to first names. All translations from Gujarati and Hindi are mine.

2 Human biology seems to favor birth of slightly fewer girls than boys, leading to newborn ratios between 0.935 and 0.971 absent outside intervention (Waldron 1998, 53–63).

3 Figure from the 2011 Census of India. The 2021 census is still pending due to COVID-19.

4 The morality and pragmatics of brokerage are central preoccupations in anthropological explorations of economic, legal, and ethical problems (Lindquist 2015a; 2015b), especially in South Asia (Björkman 2021; Huberman 2010; Perkins 2024; Zaman and van der Geest 2020). The anthropology of South Asian brokerage highlights how brokers’ ambivalent positions, actions, and ethics may illuminate contemporary conditions of rapid transformation and moral friction (Chowdhury 2019, 133; Huberman 2010, 416–20; Perkins 2024, 1–2).

5 Reproductive anthropology has long highlighted potential tensions between medical and social conceptions of procreation (Davis-Floyd 2004; Jordan 1993; Van Hollen 2003). By invoking “medical pluralism,” I am not suggesting people thought of their son production efforts exclusively as medical affairs, but rather gesturing toward how biomedicine, herbal medicine, and religion all serve as distinct modalities for striving toward the same bodily goal. It helps to remember that pragmatic resort to multiple modalities – explicitly medical or otherwise – is a response to “uncertainty about the relations, agents, and motives behind affliction” (Whyte 1997, 32).

6 Demographic research positively correlates sex selection with various markers of affluence, including maternal education, caste privilege, economic prosperity, and urban residence (Arokiasamy and Goli 2012; Kulkarni 2020, 26–27). An early-2000s field study in Mahesana District showed marked gender skewing in births to mothers having one or more girls, with disparities most pronounced among the privileged (Visaria 2007, 144–53)

7 Being a doctor-in-training helped me fit in clinically. But I also had to check my own biomedical impulses, probe doctors’ assumptions, and – most importantly – gauge how I was trespassing on intimate experiences. I always remained cognizant of the thin line between the ethnographic witness and the ethnographic vulture (Knight 2015). Moreover, I sometimes had to reassure families that I would not judge them for using non-biomedical techniques. My diasporic Gujarati identity also marked me as both insider and outsider. I spoke Gujarati fluently and understood much of local culture. At times, coupled with my paternal grandparents’ roots in a locally prosperous and powerful caste, this was enough for people to welcome me with open arms. But I was also clearly foreign – U.S.-based, a researcher, well-off, meriting both deference and skepticism. And my paternal lineage aroused suspicion from members of historically less privileged castes: Was I haughty? Was I biased? Would I write bad things about their community?

8 “Tension” is a well-established cultural idiom of feminine distress in north India, especially vis-à-vis interpersonal difficulties and contemporary stresses (Weaver 2017).

9 One of Gujarat’s first anti-sex selection campaigns targeted the herbal pill “Select” (Unnikrishnan 1993). Subsequently, national controversy flared when U.S.-based Gen-Select marketed a kit touting higher rates of son conception through nutriceuticals, diet, intercourse timing, and douching (Aravamudan 2007, 81–82). In neither case did responses confront the moral good implicit in people’s desperate resort to a technology of hope.

## References

[CR1] Allahbadia, G. N. (2002). The 50 million missing women. *Journal of Assisted Reproduction and Genetics,**19*(9), 411–416. 12408534 10.1023/A:1016859622724 PMC3455548

[CR2] Aravamudan, G. (2007). *Disappearing daughters: The tragedy of female foeticide*. Penguin Books.

[CR3] Arokiasamy, P., & Goli, S. (2012). Explaining the skewed child sex ratio in rural India: Revisiting the landholding-patriarchy hypothesis. *Economic and Political Weekley XLVII,**42*, 85–94.

[CR4] Baer, H. A. (2001). *Biomedicine and alternative healing systems in America: Issues of class, race, ethnicity, and gender*. The University of Wisconsin Press.

[CR6] Bandyopadhyay, S., & Singh, A. J. (2007). Sex selection through traditional methods in rural North India. *Indian Journal of Community Medicine,**32*, 32–34.

[CR7] Bharadwaj, A. (2006). Sacred conceptions: Clinical theodicies, uncertain science, and technologies of procreation in India. *Culture, Medicine, and Psychiatry,**30*, 451–465. 17123174 10.1007/s11013-006-9032-0

[CR8] Bharadwaj, A. (2016). *Conceptions: Infertility and procreative technologies in India*. Berghahn Books.

[CR9] Björkman, L. (2021). *Bombay brokers*. Duke University Press.

[CR10] Brodwin, Paul. 2003. *Medicine and morality in Haiti: The contest for healing power*. Cambridge University Press.

[CR11] Busby, C. (1997). Permeable and partible persons: A comparative analysis of gender and body in South India and Melanesia. *Journal of the Royal Anthropological Institute,**3*(2), 261–278.

[CR12] Busby, C. (2000). *The performance of gender: An anthropology of everyday life in a South Indian fishing village*. Routledge.

[CR13] Chowdhury, N. S. (2020). Paradoxes of the popular: Crowd politics in Bangladesh. *Stanford University Press*. 10.1515/9781503609488

[CR14] Cremers, A. (2019). Medical pluralism, boundary making, and tuberculosis in Lambaréné, Gabon. *Medicine Anthropology Theory, **6*(4). 10.17157/mat.6.4.624.

[CR17] Costa, D., Ayesha, K. V., Ryan, K., Raman, P. S., Santacatterina, M., & Mavalankar, D. (2014). The state-led large scale public private partnership ‘Chiranjeevi Program’ to increase access to institutional delivery among poor women in Gujarat, India: How has It done? What can we learn? *PLoS ONE,**9*(5), e95704. 24787692 10.1371/journal.pone.0095704 PMC4006779

[CR15] Dagar, R. (2002). *Identifying and controlling female foeticide and infanticide in Punjab. *Institute for Development and Communication.

[CR16] Davis-Floyd, R. (2004). *Birth as an American rite of passage*. University of California Press.

[CR18] Dube, L. (1986). Seed and Earth: The Symbolism of Biological Reproduction and Sexual Relations of Production. In L. Dube, E. Leacock, & S. Ardener (Eds.), *Visibility and Power: Essays on Women in Society and Development* (pp. 22–53). Oxford University Press.

[CR19] Eklund, L., & Purewal, N. (2017). The bio-politics of population control and sex-selective abortion in China and India. *Feminism & Psychology,**27*(1), 34–55. 10.1177/0959353516682262

[CR20] Evans-Pritchard, E. E. (1976). *Witchcraft, oracles, and magic among the Azande*. Clarendon Press.

[CR21] Eves, R., & Kelly-Hanku, A. (2020). Medical pluralism, Pentecostal healing and contests over healing power in Papua New Guinea. *Social Science & Medicine,**266*(December), 113381. 10.1016/j.socscimed.2020.113381 32977260 10.1016/j.socscimed.2020.113381

[CR22] Fassin, D. (2012). Toward a critical moral anthropology. In Fassin, D., ed., *A Companion to Moral Anthropology*. Wiley-Blackwell.

[CR23] Fiks, E. (2023). Care, violence, and more-than-human reproductive ecologies in North India. *Ethnos* August: 1–17. 10.1080/00141844.2023.2243398.

[CR24] Flueckiger, J. B. (2017). *When the World Becomes Female: Guises of a South Indian Goddess.* Indiana University Press.

[CR25] Franklin, S. (1997). *Embodied progress: A cultural account of assisted conception*. Routledge.

[CR26] Franklin, S. (2006). Origin stories revisited: IVF as an anthropological project. *Culture, Medicine and Psychiatry,**30*(4), 547–555. 10.1007/s11013-006-9036-9 17120156 10.1007/s11013-006-9036-9

[CR27] Galvin, M., Michel, G., Manguira, E., Pierre, E., Lesorogol, C., Trani, J.-F., Lester, R., & Iannotti, L. (2023). Examining the etiology and treatment of mental illness among vodou priests in Northern Haiti. *Culture, Medicine, and Psychiatry,**47*(3), 647–668. 10.1007/s11013-022-09791-4 35753013 10.1007/s11013-022-09791-4 PMC9244373

[CR28] Gammeltoft, T. (2002). Between ‘Science’ and ‘Superstition’: Moral perceptions of induced abortion among young adults in Vietnam. *Culture, Medicine, and Psychiatry,**26*, 313–338. 12555903 10.1023/a:1021210405417

[CR29] Good, B. (1994). *Medicine, rationality, and experience: An anthropological perspective*. Cambridge University Press.

[CR30] Good, B. J., Del Vecchio Good, M.-J., Togan, I., Ilbars, Z., Güvener, A., & Gelişen, I. (1994). In the subjunctive mode: Epilepsy narratives in Turkey. *Social Science & Medicine,**38*(6), 835–842. 10.1016/0277-9536(94)90155-4 8184334 10.1016/0277-9536(94)90155-4

[CR31] Goslinga, G. (2011). Embodiment and the metaphysics of virgin birth in South India: A case study. In A. Dawson (Ed.), *Summoning the spirits: Possession and invocation in contemporary religion* (pp. 109–123). I.B. Tauris.

[CR32] Government of Gujarat. (2009). *Annual Administrative Report: 2008-2009.* Gandhinagar: Gujarat Commissionerate of Health, Medical Services and Medical Education.

[CR33] Guilmoto, C. (2015). The masculinization of births: Overview and current knowledge. *Population,**70*(2), 185–242.

[CR34] Halliburton, M. (2023). Hegemony versus pluralism: Ayurveda and the movement for global mental health. *Anthropology & Medicine,**30*(2), 85–102. 10.1080/13648470.2020.1785842 32873052 10.1080/13648470.2020.1785842

[CR35] Hampshire, K. R., & Owusu, S. A. (2013). Grandfathers, Google, and Dreams: Medical pluralism, globalization, and new healing encounters in Ghana. *Medical Anthropology,**32*(3), 247–265. 10.1080/01459740.2012.692740 23557008 10.1080/01459740.2012.692740

[CR36] Hollenberg, D., & Muzzin, L. (2010). Epistemological challenges to integrative medicine: An Anti-colonial perspective on the combination of complementary/alternative medicine with biomedicine. *Health Sociology Review,**19*(1), 34–56. 10.5172/hesr.2010.19.1.034

[CR37] Hsu, E. (2008). Medical pluralism. In K. Heggenhougen & S. Quah (Eds.), *International Encyclopedia of Public Health* (pp. 316–321). Elsevier.

[CR38] Huberman, J. (2010). The Dangers of Dalālī, the Dangers of Dān. *South Asia,**33*(3), 399–420.

[CR39] IIPS. (2010). *District Level Household and Facility Survey (DLHS-3), 2007-08: Gujarat.* International Institute for Populations Sciences.

[CR40] Inhorn, M. (1994). *Quest for conception: Gender, infertility, and Egyptian medical traditions*. University of Pennsylvania Press.

[CR41] Inhorn, M. (2015). *Cosmopolitan conceptions: IVF sojourns in Global Dubai*. Duke University Press.

[CR42] Jeffery, P., Jeffery, R., & Lyon, A. (1989). *Labour pains and labour power: Women and childbearing in India*. Zed Books.

[CR43] John, M., Kaur, R., Palriwala, R., Raju, S., Sagar, A. (2008). *Planning families, planning gender.* ActionAid and IDRC.

[CR44] Jordan, B. (1993). *Birth in four cultures: A crosscultural investigation of childbirth in Yucatan, Holland, Sweden, and the United States*. Waveland.

[CR45] Kaur, R., Bhalla, S., Agarwal, M., & Ramakrishnan, P. (2016). *Sex Ratio Imbalances and Marriage Squeeze in India: 2000-2050.* UNFPA.

[CR46] Kaur, R., & Kapoor, T. (2021). The gendered biopolitics of sex selection in India. *Asian Bioethics Review,**13*(1), 111–127. 10.1007/s41649-020-00159-7 33717350 10.1007/s41649-020-00159-7 PMC7813894

[CR47] Kaur, R., & Palriwala, R., eds. (2014). *Marrying in South Asia: Shifting concepts, changing practices in a globalising world*. New Delhi: Orient Blackswan.

[CR48] Khanna, S. (2009). *Fetal/fatal knowledge: New reproductive technologies and family-building strategies in India*. Cengage Learning.

[CR49] King, D., & Stone, L. (2010). Lineal masculinity: Gendered memory within patriliny. *American Ethnologist,**37*(2), 323–336.

[CR50] Kleinman, A. (1988). *The illness narratives: Suffering, healing, and the human condition*. Basic Books. 10.1097/ACM.0000000000001864 28952997

[CR51] Knight, K.R. (2015). *Addicted.Pregnant.Poor*. Duke University Press.

[CR52] Kołodziejska-Degórska, I. (2016). Patients’ webs of relations in the medical landscapes of Central Ukraine. *Anthropology & Medicine,**23*(2), 155–171. 27362484 10.1080/13648470.2016.1180583

[CR53] Kulkarni, P. (2020). *Sex ratio at birth in India: Recent trends and patterns.* UNFPA.

[CR54] Laidlaw, J. (2014). The subject of virtue: An anthropology of ethics and freedom. Cambridge University Press.

[CR55] Lamb, S. (1997). The making and unmaking of persons: Notes on aging and gender in North India. *Ethos,**25*(3), 279–302. 10.1525/eth.1997.25.3.279

[CR56] Lamb, S. (2018). Being single in India: Gendered identities, class mobilities, and personhoods in flux. *Ethos,**46*(1), 49–69. 10.1111/etho.12193

[CR57] Lambert, H. (2012). Medical Pluralism and medical marginality: Bone doctors and the selective legitimation of therapeutic expertise in India. *Social Science & Medicine,**74*(7), 1029–1036. 10.1016/j.socscimed.2011.12.024 22336733 10.1016/j.socscimed.2011.12.024

[CR58] Lang, C., & Halliburton, M. (2023). Curiosity and creative experimentation among psychiatrists in India. *Culture, Medicine, and Psychiatry,**48*(2), 310-328. 10.1007/s11013-023-09829-1 37713142 10.1007/s11013-023-09829-1 PMC11217086

[CR59] Langford, J. (1999). Medical mimesis: Healing signs of a cosmopolitan ‘quack.’ *American Ethnologist,**26*(1), 24–46.

[CR60] Last, M. (1981). The Importance of Knowing about not knowing. *Social Science & Medicine,**15*(3), 387–392. 10.1016/0160-7987(81)90064-8 7313724 10.1016/0160-7987(81)90064-8

[CR61] Leslie, C. (1980). Medical pluralism in world perspective. *Social Science & Medicine,**14*(4), 191–195. 10.1016/0160-7987(80)90044-7 7209590 10.1016/0160-7987(80)90044-7

[CR62] Lindquist, J. (2015a). Brokers and brokerage, Anthropology of. In *International Encyclopedia of the Social & Behavioral Sciences (Second Edition)*, edited by James D. Wright, 870–874. Oxford: Elsevier. 10.1016/B978-0-08-097086-8.12178-6.

[CR63] Lindquist, J. (2015b). Of figures and types: brokering knowledge and migration in Indonesia and beyond. *Journal of the Royal Anthropological Institute* 21 (S1): 162–177. 10.1111/1467-9655.12172.

[CR64] Livingston, J. (2012). *Improvising medicine: An African oncology ward in an emerging cancer epidemic*. Duke University Press. 10.1080/13648470.2014.929634 24962205

[CR65] Lukšaitė, E. (2022). ‘I Do Not Have to Hurt My Body Anymore’: Reproductive chronicity and sterilization as ambivalent care in rural North India. *Medical Anthropology Quarterly,**36*(3), 312–328. 10.1111/maq.12709 35524762 10.1111/maq.12709 PMC9545858

[CR66] Majumdar, A. (2023). Infertility as inevitable: Chronic lifestyles, temporal inevitability and the making of abnormal bodies in India. *Anthropology & Medicine,**30*(2), 120–134. 10.1080/13648470.2021.1874872 34256670 10.1080/13648470.2021.1874872

[CR67] Mattingly, C. (1994). The concept of therapeutic ‘emplotment.’ *Social Science & Medicine,**38*(6), 811–122. 10.1016/0277-9536(94)90153-8. 10.1016/0277-9536(94)90153-8 8184332

[CR68] Mattingly, C. (2010). *The paradox of hope: Journeys through a clinical borderland*. University of California Press.

[CR69] Neogi, S. B. (2021). *Gender before birth in India: Role of indigenous and traditional medicines*. Springer. 10.1007/978-981-16-3318-8.

[CR70] Obermeyer, C. M. (2000). Pluralism and Pragmatism: Knowledge and practice of birth in Morocco. *Medical Anthropology Quarterly,**14*(2), 180–201. 10.1525/maq.2000.14.2.180 10879369 10.1525/maq.2000.14.2.180

[CR71] Ong, A. (1988). The production of possession: Spirits and the multinational corporation in Malaysia. *American Ethnologist,**15*(1), 28–42.

[CR72] Ortner, S. (2016). Dark anthropology and its others: Theory since the eighties. *HAU,**6*(11), 47–73.

[CR73] Pandian, A., & Ali, D. (2010). Introduction. In A. Pandian & D. Ali (Eds.), *Ethical life in South Asia* (pp. 1–18). Indiana University Press.

[CR74] Paxson, H. (2006). Reproduction as spiritual kin work: Orthodoxy, IVF, and the moral economy of motherhood in Greece. *Culture, Medicine, and Psychiatry,**30*, 481–505. 17051429 10.1007/s11013-006-9028-9

[CR75] Penkala-Gawęcka, D., and M. Rajtar. (2016). Introduction to the special issue ‘Medical Pluralism and Beyond.’ *Anthropology & Medicine**23*(2), 129–34. 10.1080/13648470.2016.1180584. 10.1080/13648470.2016.1180584 27464971

[CR76] Perkins, J. E. (2024). “‘Beware of *dalals*’: A moral world of health market brokerage in Bangladesh.” *Journal of the Royal Anthropological Institute*, *30*, 589-607. 10.1111/1467-9655.14101.

[CR77] Petryna, A. (2002). *Life exposed: Biological citizens after Chernobyl*. Princeton University Press.

[CR78] Pinto, S. (2008). *Where There Is No Midwife: Birth and Loss in Rural India*. Berghahn Books.

[CR79] Pinto, S. (2015). ‘The Tools of Your Chants and Spells’: Stories of madwomen and Indian practical healing. *Medical Anthropology,**35*(3), 263–277. 26263046 10.1080/01459740.2015.1081902

[CR80] Prasad, P. (2007). Healing in South Gujarat: Conceptions, practices and restricted medical pluralism. *Indian Anthropologist,**37*(1), 13–28.

[CR81] Purewal, N. (2014). Disciplining the sex ratio: Exploring the governmentality of female foeticide in India. *Identities,**21*(5), 466–480.

[CR82] Purewal, N. (2018). Sex selective abortion, neoliberal patriarchy and structural violence in India. *Feminist Review,**119*(1), 20–38. 10.1057/s41305-018-0122-y

[CR83] Raffaetà, R., Krause, K., Zanini, G., & Alex, G. (2017). Medical pluralism reloaded. *L’uomo,**1*, 95–124. 10.7386/89101

[CR84] Robbins, J. (2013). Beyond the suffering subject: Toward an anthropology of the good. *Journal of the Royal Anthropological Institute,**19*, 447–462.

[CR85] Roberts, E. (2012). *God’s laboratory: Assisted reproduction in the Andes*. University of California Press.

[CR86] Roberts, E. (2016). Gods, germs, and petri dishes: Toward a nonsecular medical anthropology. *Medical Anthropology, **35*(3), 209–219. 10.1080/01459740.2015.1118100 26930040

[CR87] Samuels, A. (2018). ‘This Path Is Full of Thorns’: Narrative, subjunctivity, and HIV in Indonesia. *Ethos,**46*(1), 95–114. 10.1111/etho.12194

[CR88] Sandesara, U. (In submission). The narrative governance of life: Morality, melodrama, and the limits of biopower in Western Indian efforts against sex selection. 10.1111/maq.12901 PMC12140136 39612345

[CR89] Singh, B. (2015). *Poverty and the quest for life: Spiritual and material striving in rural India*. University of Chicago Press.

[CR90] Stork, H. (1992). Mothering rituals in Tamilnadu: Some magico-religious beliefs. In J. Leslie (Ed.), *Roles and rituals for Hindu Women* (pp. 89–106). Motilal Banarsidass.

[CR91] Street, A. (2014). *Biomedicine in an unstable place: Infrastructure and personhood in a Papua New Guinean hospital*. Duke University Press. 28218947

[CR107] Tong, Y. (2022). *India's Sex Ratio at Birth Begins to Normalize*. Pew Research Center.

[CR92] Turner, V. (1967). A Ndembu doctor in practice. *The forest of symbols: Aspects of Ndembu rituals* (pp. 359–393). Cornell University Press.

[CR93] Unnikrishnan, P. V. (1993). Banned: Select, a drug to alter the sex of the foetus. *Health for the Millions,**1*(2), 29–30. 12286470

[CR94] Unnithan-Kumar, M. (2004). Conception technologies, local healers and negotiations around childbearing in Rajasthan. In M. Unnithan-Kumar (Ed.), *Reproductive agency, medicine, and the state: Cultural transformations in childbearing* (pp. 59–81). Berghahn Books.

[CR95] Unnithan-Kumar, M. (2010). Learning from infertility: Gender, health inequities and faith healers in women’s experiences of disrupted reproduction in Rajasthan. *South Asian History and Culture,**1*(2), 315–327.

[CR96] Unnithan-Kumar, M. (2011). Female selective abortion – beyond ‘culture’: Family making and gender inequality in a globalising India. *Culture, Health, and Sexuality,**12*, 153–166. 10.1080/13691050902825290 19437177

[CR5] van Balen, F., & Inhorn, M. (2003). Son preference, sex selection, and the 'new" new reproductive technologies. *International Journal of Health Services,**33*(2), 235–252. 12800886 10.2190/PP5X-V039-3QGK-YQJB

[CR97] Van Hollen, C. (2003). *Birth on the threshold: Childbirth and modernity in South India*. University of California Press.

[CR98] Visaria, L. (2007). Sex-selective abortion in Gujarat and Haryana: Some empirical evidence. In L. Visaria & V. Ramachandran (Eds.), *Abortion in India: ground realities* (pp. 133–167). Routledge.

[CR99] Wahlberg, A., & Gammeltoft, T. (2018). *Selective reproduction in the 21st century*. Palgrave Macmillan.

[CR100] Waldron, I. (1998). *Too young to die: Genes or gender?* UN Population Division.

[CR101] Weaver, L. J. (2017). Tension among women in North India: An idiom of distress and a cultural syndrome. *Culture, Medicine, and Psychiatry,**41*(1), 35–55. 10.1007/s11013-016-9516-5 28050759 10.1007/s11013-016-9516-5

[CR102] Whitaker, E. D. (2003). The idea of health: History, medical pluralism, and the management of the body in Emilia-Romagna, Italy. *Medical Anthropology Quarterly,**17*(3), 348–375. 10.1525/maq.2003.17.3.348 12974202 10.1525/maq.2003.17.3.348

[CR103] Whittaker, A. (2004). *Abortion, sin and the state in Thailand*. Routledge Curzon.

[CR104] Whyte, S. R. (1997). *Questioning misfortune: The pragmatics of uncertainty in Eastern Uganda*. Cambridge University Press.

[CR105] Zaman, S., & van der Geest, S. (2020). Brokers on the ward: ward boys, cleaners, and Gatemen in a Bangladeshi hospital. *Asian Journal of Social Scence,**48*(1-2), 92–114. 10.1163/15685314-04801006

[CR106] Zhang, E. Y. (2007). Switching between Traditional Chinese Medicine and Viagra: Cosmopolitanism and Medical Pluralism Today. *Medical Anthropology,**26*(1), 53–96. 10.1080/01459740601184448 17365637 10.1080/01459740601184448

